# Supplementary material for: Complex‐centric proteome profiling by SEC‐SWATH‐MS
Source: Mol Syst Biol. 2019 Jan 14;15(1):e8438. doi: 10.15252/msb.20188438 (PMC6346213; doi:10.15252/msb.20188438)
Supplement: Supplementary file 7 — Dataset EV6 [file MSB-15-e8438-s007.zip › feature_plots_bioplex/Q9H6Y5.pdf]

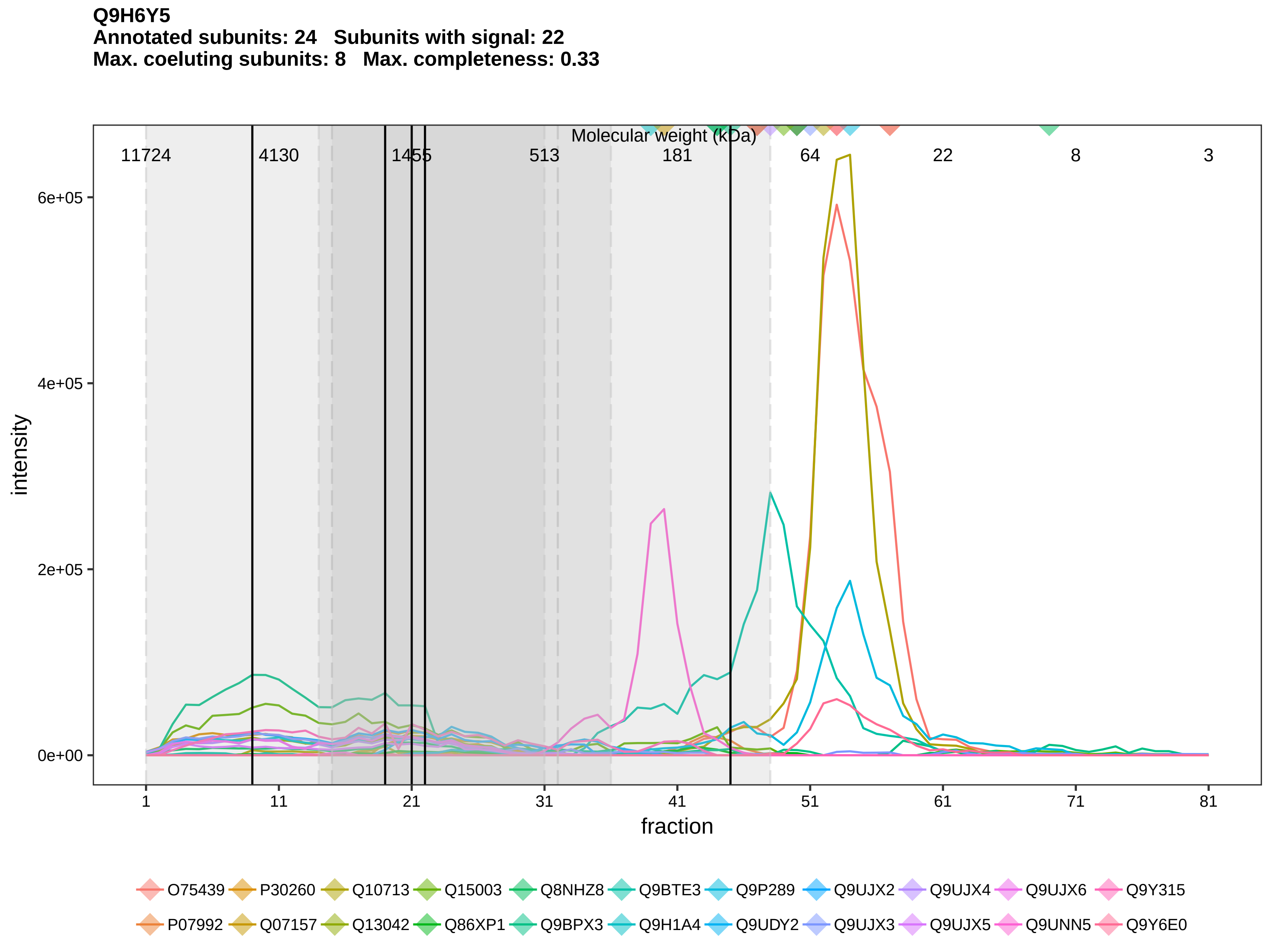

Q9H6Y5
Annotated subunits: 24 Subunits with signal: 22
Max. coeluting subunits: 8 Max. completeness: 0.33
Molecular weight (kDa)
11724
4130
1455
513
181
64
22
8
3
intensity
6e+05
4e+05
2e+05
0e+00
1
11
21
31
41
51
61
71
81
fraction
O75439
P30260
Q10713
Q15003
Q8NHZ8
Q9BTE3
Q9P289
Q9UJX2
Q9UJX4
Q9UJX6
Q9Y315
P07992
Q07157
Q13042
Q86XP1
Q9BPX3
Q9H1A4
Q9UDY2
Q9UJX3
Q9UJX5
Q9UNN5
Q9Y6E0
